# Exergaming to Increase Physical Activity in Older Adults: Feasibility and Practical Implications

**DOI:** 10.1007/s11897-024-00675-9

**Published:** 2024-07-18

**Authors:** Patrik Rytterström, Anna Strömberg, Tiny Jaarsma, Leonie Klompstra

**Affiliations:** 1 Department of Health, Medicine and Caring Sciences, Linköping University, Linköping, Sweden; 2 Department of Cardiology, Linköping University, Linköping, Sweden

**Keywords:** Exergame, Active video game, Elderly, Exercise, Physical activity, Virtual reality

## Abstract

**Purpose of Review:**

To evaluate the feasibility of exergaming among older adults, focusing on acceptability, demand, implementation, and practicality. Additionally, to offer practical implications based on the review's findings.

**Recent Findings:**

Exergaming is a safe for older adults, potentially increasing physical activity, balance, cognition, and mood. Despite these possible benefits, barriers such as unfamiliarity with equipment, complex controls, and unclear instructions may challenge older adults in exergaming.

**Summary:**

Based on the experience of older adults, they found exergaming enjoyable, particularly the social interactions. Exergaming was perceived as physically and cognitively demanding, with technical and safety challenges. Introducing exergaming requires thorough familiarization, including written and video instructions, follow-up support, and home accessibility. To be able to follow improvements during exergaming as well as age-appropriate challenges are important for successful integration into daily life.

Based on these findings, an ExerGameFlow model for older adults was developed which provides practical implications for future design of exergames and interventions.

**Supplementary Information:**

The online version contains supplementary material available at 10.1007/s11897-024-00675-9.

## Introduction

There is a growing recognition of the importance of maintaining an active lifestyle to promote physical activity in all age groups [1]. Recent studies, suggest that exercising with virtual reality technology, such as exergaming, has the potential to improve physical activity in a range of individuals, including older adults [2].

Exergames (a combination of exertion and video games including strength training, balance, aerobic endurance and flexibility activities) [3, 4] are promising for increasing physical activity engagement. Systematic literature reviews show that exergaming is safe and feasible in older adults, could increase motivation in becoming more physically active, could increase physical activity, balance and cognition, could decrease depressive symptoms and could facilitate healthy aging [5–12]. A systematic review and meta-analysis showed that exergaming has an effect equivalent to other types of exercising on improving walking in older adults [8].

While exergaming holds potential benefits, older adults might feel unsure or discouraged to exergaming because of unfamiliar equipment, new physical movements, complicated controls, confusing displays, unclear instructions in the game or short familiarization to the exergame, and they can feel that the exergames are too fast for them to play [13–18].

Although evidence show benefits of exergaming in older adults, more information on the feasibility of exergames in older adults, in terms of the usability and acceptance of exergames in this group, could be useful for future design of exergames and interventions including exergames. Therefore, the aim of this systematic literature review was to assess the feasibility of exergaming in older adults based on the experiences of acceptability, demand, implementation, and practicality of exergames. If older adults do not enjoy exergaming, they will be less likely to play the game. A model that consists of a set of criteria that can be used to design and evaluate games with respect to player enjoyment is the GameFlow model [54]. Although the GameFlow model is applicable to most game genres and platforms, this model us not used for exergames or for gaming in older adults. Therefore, we found that the model might need some adaptation to be used design exergames for older adults. Therefore, the second aim of this study was to provide practical implications for exergaming in older adults based on the results of the literature review by adapting the GameFlow model [54].

## Method

### Design

This study is a systematic literature review based on studies with a qualitative design.

### Literature Search

The initial search started in PubMed for identification of primary studies. A text analysis was carried out with these articles titles and abstracts and index terms identified and was applied to four online electronic databases (PubMed, Scopus, CINAHL and Web of Science), and was conducted in January 2024. The following search string was utilized across the databases: ((exergames OR exergaming OR active AND video AND games) AND (experiences OR perceptions OR attitudes OR views) AND (qualitative AND research OR qualitative AND study OR qualitative AND methods OR interview) AND (elderly OR aged OR older OR elder OR geriatric)).

For inclusion in this analysis, studies were required two be published or in press, peer-reviewed literature and in the English language. Studies that did not include older adults (aged ≥ 65 years), studies that were not explicitly related to physical activity, and studies that were not specifically related to experience in exergaming were excluded. Additionally, we excluded literature reviews, protocols, and case-studies.

The study was registered at PROSPERO International prospective register of systematic reviews: CRD42022360720.

### Selection of Studies

**Fig. 1:**
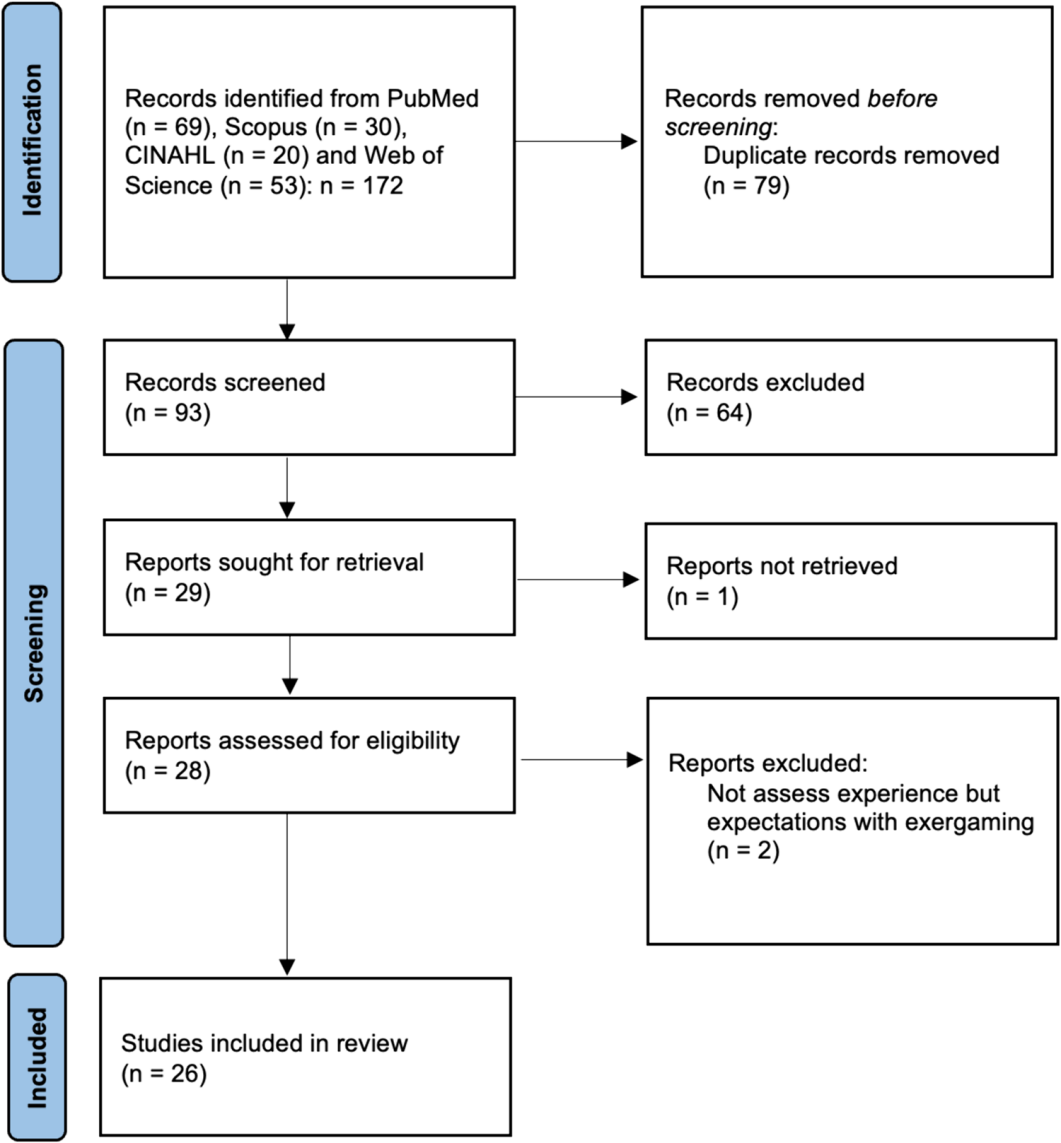
Flowchart for the articles included in the qualitative systematic review

In total 93 articles were identified by the databases, whereof 67 articles were excluded from the analysis. One article was not possible to retrieve, and two articles were focussing on expectations of exergaming and not on the experience of older adults with exergaming. In total 26 were included in this qualitative systematic literature review (Fig. 1). To strengthen the content validity, two authors (LK & PR) independently reviewed the results of each search according to the inclusion and exclusion criteria. Discussions on disagreements receded the final decision on inclusion.

### Quality Appraisal of the Studies

For quality appraisal, the Critical Appraisal Skill Programme (CASP UK, 2020) systematic checklist were used to evaluate the rigour of studies (Supplement Table 1). Irrespective of the assessment criteria used to evaluate the quality of the methods in the articles, all relevant studies in the emerging field of exergaming were incorporated if they were deemed to have meaningful results. The quality appraisal serves to demonstrate strengths and weaknesses in method and substance (Supplement Table 1). All articles were summarized based on relevant information about title, authors, settings, method, authors conclusions (Table 1).

**Table 1:** Description of the articles included in the systematic review on feasibility of exergaming in older adults

	Author, year, country and publication type	Aim of the qualitative data collection	Exergame used Sessions/time	Home/research facility	Stated method	Sample size/age/sex	The qualitative results
1	Agmon et al., 2011, USA, journal article	To determine the safety and feasibility of exergaming to improve balance among older adults with balance impairments in a 3-month individualized home-based training program	Nintendo Wii Fit, 30 min 3 times per week for 3 months	Home	Telephone calls, exit interviews Content analysis of home visit logs,	11 older adults, 4 females	Use of Wii Fit for limited supervised balance training in the home was safe and feasible for a selected sample of older adults. Further research is needed to determine clinical efficacy in a larger, diverse sample and ascertain whether Wii Fit exergames can be integrated into physical therapy practice to promote health in older adults
2	Barg-Walkow et al., 2017 USA, journal article	To identify key barriers and facilitators to exergame use	Microsoft Xbox 360 with Kinect system One-session	Research facility	Interviews Thematic analysis approach	23 older adults, 13 females	The older adults were open to adopting exergames, which could provide opportunities to increase physical activity
3	Brown et al.,2015 USA, journal article	To qualitatively describe 1) upper extremity use at home, 2) previous home exercise or activity programs, and 3) the accept- ability of a novel upper extremity home program	NeuroGame Therapy	At home	Interviews A qualitative descriptive approach	10 patients with stroke, 4 females	NeuroGame was reported to be an acceptable home intervention, with perceived effectiveness
4	Cacciata et al., 2021 USA, journal article	To explored facilitators and challenges using a home-based exergame platform, the Nintendo Wii Sports, in patients with heart failure	Nintendo Wii Sports	At home 30 min a day for 3 months	Interviews Content analysis	13 patients with heart failure, 5 females	Exergaming can increase individuals' physical activity because of easy accessibility and the fun and motivating factors the games offer. Participants initially found exergaming enjoyable and challenging. However, engagement diminished over time because of boredom from playing the same games for a period of time
5	Celinder et al., 2012 Europe, journal article	To explore stroke patients’ experiences with exergaming as a supplement to conventional occupational therapy in a controlled hospital setting	Nintendo Wii Sports	In hospital setting Between 1–9 sessions for 3 weeks	Interviews and field notes Content analysis,	9 patients with stroke, 3 females	Stroke patients in hospital settings may experience exergaming as a beneficial and challenging occupation for both rehabilitation and leisure
6	Cao et al., 2016 USA, journal article	To understand the facilitators and barriers to exercise using exergames among assisted living residents, particularly in the area of cognitive, physical, and psychosocial effects	NIntend Wii Fit	Assisted living facility	Interviews Content Analysis	15 older adults, 11 females	An intervention incorporating self-efficacy and Wii exergames did result in the identification of cognitive, physical, and psychosocial benefits and barriers to exercise
7	Chu et al., 2021 Canada, journal article	To identify facilitators and barriers to the implementation of our exergaming intervention into LTC homes according to staff input	the MouvMat	Long-term care homes	Interviews, observational logs and field notes Content analysis	28 older adults, 13 long-term care home residents, and 15 staff and family members	residents enjoyed engaging with the prototype and appreciated the opportunity to increase their PA. In addition, staff and stakeholders were drawn to the exergame ability to increase residents’ autonomous PA. The intended and perceived benefits of exergaming, that is, improved physical and cognitive health, were the most common facilitators of its use identified by study participants
8	Demers et al., 2018 Canada, journal article	To determine user satisfaction and safety of incorporating a low-cost virtual rehabilitation intervention as an adjunctive therapeutic option for cognitive-motor upper limb rehabilitation in individuals with sub-acute stroke	Smask Blocks game, Shoppers’ Delight game	Rehabilitation setting 1 single session	Focus groups and interviews Thematic content analysis	7 patients with stroke, 2 females	Three main themes emerged from the patient interviews: Perceived usefulness in rehabilitation, satisfaction with the virtual reality intervention and aspects to improve
9	De Vries et al., 2018 Europe, journal article	To study which games and underlying game mechanics are considered motivating by older adults	Nintendo Wii sports resort Microsoft Xbox 360 with Kinect system Sports	Sports facility	interviews UX-laddering	30 older adults, 20 females	Game speed should be fast enough to increase challenge, but not so fast that it compromises the value ‘‘Stay Relaxed.’’ This emphasizes the importance of games to be adaptable to the skills of the player
10	Ellmers et al., 2018 Europe, journal article	To assess perceived changes in psychological and physical functioning	PONG	8 sessions in 4 weeks in the communal living areas	Focus groups Thematic analysis	26 older adults, 21 females	The improvements could be a consequence of changes in perceived action capabilities, with participants stating that they were more “aware” of their balance abilities This increased awareness resulted in a number of participants reporting increased confidence, with these participants developing the confidence needed to attempt more challenging balance tasks in daily life. We propose that post-intervention improvements in postural control may have been the result of a ‘recalibration’ of participants’ perception of their balance capabilities
11	Finley & Combs, 2013 USA, journal article	To utilize input from focus groups of gaming intervention users with chronic stroke to identify characteristics of gaming that influence user/patient engagement in the activity	In the Groove Dancetown	1 session	Focus groups and guided group interviews No analyses method describes for qualitative data	10 patients with stroke, 4 females	Participants enjoyed playing the gaming systems. Three primary themes emerged differentiating the systems: (1) musical encouragement; (2) focus and attention; and (3) motivation provided by performance feedback
12	Forsberg et al., 2015 Europe, journal article	To describe the experiences of exergaming for balance exercise, from the perspective of patients with multiple sclerosis	Nintendo Wii Fit	Hospital setting. 12 individual sessions of 30 min of balance exercise twice a week	Interviews Content analysis	15 patients with multiple sclerosis, 9 females	Patients said that exercising with Wii Fit games was fun, and that it challenged their physical and cognitive capacities. The competitive content in the games provided motivation to continue playing. Patients reported improved body control and, more importantly, positive effects on balance and walking in daily life. The patients regarded Wii training as a possible home training solution
13	Freed et al., 2021 USA, journal article	To investigate older adults’ perceptions of two commercially available exergames	Microsoft Xbox 360 with Kinect system: Just Dance and Kinect Sports Rivals	Lab visit. 1 session	Qualitative feedback on exergaming No analyses method describes for qualitative data	20 older adults, 16 females	The preliminary results of this pilot study suggest that exergames may help address social isolation and loneliness—particularly during times of social distancing
14	Howes et al., 2021 Europe, journal article	To explore older adults’ experience using an exergame system designed to deliver falls prevention exercise	Alienware PC connected to a Microsoft Kinect camera	Centre visit 6 × 1-h sessions	Observations and qualitative feedback The Technology Acceptance Model	7 older adults	Older adults’ experience with the system was influenced by physical health changes associated with ageing. Overall feedback after using the system was positive. Social support, from either the clinician or a peer, was a key theme influencing experience
15	Glännfjord et al., 2017 Europe, journal article	To examine how the use of the Wii Sports Bowling in an activity group was perceived by elderly people	Nintendo Wii Sports Bowling	Center for elderly people	Observations and interviews Content analysis	6 older adults, 1 female	The exergame was described as easier to play compared to real-life bowling and was enjoyable and a social activity. The opportunity to meet the group each week was important for the participants. Playing the game resulted in signs of immersion and a flow-like state. The exergame was perceived to be easy to use, to provide a way to socialize with peers and to give opportunities to participate in activities in a new way
16	Klompstra et al., 2017 Europe, journal article	Describe the experiences of patients with Heart failure when using an exergame platform at home	Nintendo Wii with the games Wii sports	At home	Semi-structured interviews Content analysis	14 patients with heart failure (6 women, ages ranging between 56 and 81 years)	Knowing the sports in the game in real life helped patients to select a specific game to play. Exergaming was an activity that added value in terms of improvement of their physical and/or mental state. Some described feeling too tired or experienced exergaming as boring as a reason for not wanting to exergame
17	Koh et al., 2020 Autralia, journal article	Explore participants’ and physiotherapists’ experiences regarding the acceptability, implementation, and practicality of a novel group-based multifactorial falls prevention activity programme for community-dwelling older people after stroke	MIRArehab	A health and wellness centre of a university	A semi-structured interview Thematic analyses	5 patients with stroke over the age of 50	Running the programme was feasible. Participants experienced positive changes in mental, emotional and social well-being
18	Meekes & Stanmore, 2017 Europe, journal article	Determine the factors that may influence the motivation of older people to use exergames to improve their physical function and reduce fall risk	MIRA Rehab	Assisted living facilities	Semi-structured interviews & observations Constant comparative method	12 older adults, 6 females	The participants were intrinsically motivated to participate in the exergames because of the enjoyment experienced when playing the exergames and perceived improvements in their physical and mental health and social confidence. The social interaction provided in this study was an important extrinsic motivator that increased the intrinsic motivation to adhere to the exergame program
19	Millington, 2015 Canada, journal article	Understanding how games such as Wii Bowling are being put to use in retirement centre contexts and the implications of such activity	Nintendo Wii	Retirement centres in the province of Ontario, Canada. Residents in the ‘independent living’ sections of these facilities	Semi-structured Interviews & observations Etnography	8 older adults, no number of females available	Exergames are deemed valuable in the process of promoting both social engagement and physical activity. It can bring people together in communal spaces while also ‘getting them up’ and active Exergaming presents challenges For retirement centre residents, it engenders health risks while also demanding the deft synchronization of media and physical literacies. For activities coordinators and other members of staff responsible for residents' care, it means they too must stay abreast of the technology sector's latest innovations; they must develop media and physical literacies of their own
20	Money et al., 2019 Europe, journal article	Exploring game usability and older adults’ perceptions and attitudes towards using the game Falls Sensei in practice	Falls Sensei, a first-person 3D exploration game	University campus	Think aloud and telephone interviews Deductive and inductive thematic analysis	15 older adults between 50 and 80 years old, 9 females	Game could be useful for everyone, as a re- minder of safe practice within the home. The game experienced as a positive learning experience despite difficulties with instructions and controls. There was evidence of increased safety awareness and a fear that someone else may be hurt as a result of a falls hazard
21	Rand et al., 2018 Asia, journal article	Explores the experiences and perceptions of individuals with chronic stroke who participated in a novel community-based video-game group intervention and their therapists	Microsoft Xbox Kinect, Sony PlayStation 2 Eyetoy, Sony PlayStation 3 MOVE, and Nintendo Wii Fit. In addition, the SeeMe VR system (Virtual Reality Kinect Rehabilitation	Rehabilitation centre	Semi-structured interviews and a focus group interview Content analysis	8 patients with stroke, 4 females	Three main categories were identified by the study participants: (a) using video games, (b) the group/team experience, and (c) intervention outcomes/evolving understandings following the intervention. Playing videogames was perceived not as treatment but as a motivating tool to facilitate whole-body movement
22	Rogers et al., 2021 Africa, journal article	explore participants’ constructs of falls and fall prevention. In addition, participants were invited to share their experiences, in- cluding barriers and facilitators regarding adherence, with the WBB exergaming intervention	Wii Fit Balance Board	Residential communities of retirement apartments	Focus group interviews Content analysis	15 older adults, 2 females	Attitudes toward falls and fall prevention were explored, as well as participants’ experiences of the exergaming programme. Consistent with a developing country con- text, participants acknowledged both intrinsic and extrinsic fall risk factors
23	Swinnen et al., 2021 Europe, journal article	investigate the usability of the VITAAL exergame prototype through a mixed methods design in institutionalized older adults with MNCD	VITAAL individualized multicomponent stepping exergame training solution for geriatric rehabilitation	Long-term care facility	A semi-structured interview, field notes and think aloud method Thematic analysis	22 patients with major neurocognitive disorder, 18 females	Result showed five main themes which describe the experiences of the participants: perceived user friendliness and acceptability of the exergames; interactional experience; motivational factors; training; and risks
24	Tabak et al., 2020 Europe, journal article	Older adults’ experiences using game-based, mobile coaching application compared with a standard coaching application in terms of engagement, motivation to be physically active, and in relation to the applied design features	ActivityCoach for 1 week WordFit for up to 3 weeks	At home	Interviews Content analysis	20 older adults, 10 females	The participants’ experience is described from two perspectives: motivation to be physically active and engagement in using the applications
25	Valenzuela et al., 2018 Australia, journal article	(1) to explore older adults’ experiences using SureStep, an interactive cognitive-motor step training program to reduce fall risk unsupervised at home; (2) to explore which program features older adults considered encouraged program uptake and adherence; (3) to identify usability issues encountered by older adults when using the technology independently at home; and (4) to provide guidance for the design of a future technology-based exercise program tailored to older adults to use independently at home as a fall prevention strategy	SureStep consists of 4 games (StepMania, Stepper, Trail-stepping, and Tetris) that are modified versions of popular video games	At home	Structured interviews with open-ended questions Thematic analysis	24 older adults, 17 females	Findings suggest older adults are open to use technology-based exercise programs at home, and in the context of optimizing adherence to home-based exercise programs for the prevention of falls, findings suggest that program developers should develop exercise programs in ways that provide older adults with a fun and enjoyable experience (thus increasing intrinsic motivation to exercise), focus on improving outcomes that are significant to older adults (thus increasing self-determined extrinsic motivation), offer challenging yet attainable exercises (thus increasing perceived self-competence), provide positive feedback on performance (thus increasing self-efficacy), and are easy to use (thus reducing perceived barriers to technology use)
26	Vaziri et al., 2016 Europe, journal article	To iden- tify factors influencing usability, user experience and user acceptance of older adults engaging with an ICT- based fall prevention system (iStoppFalls)	iStoppFalls system including 6 technical components	Home	Semi-structured interviews Content analytic approach	40 older adults, number of females not available	Interview data reported different and often contradiction experiences related to usablity, user experience and user acceptance

### Analysis of Studies

All of the included articles were categorised using a qualitative deductive content analysis [19], based on Bowen´s four areas of focus of feasibility [20]: (1) Acceptability, how the participants reacted to the exergaming; (2) Demand, demand of exergaming; (3) Implementation of exergaming, extend, likelihood, and the way exergaming can be implemented in the participants life) (4) Practicality, the extent to which an exergaming can be delivered when resources, time, commitment, or some combination thereof are constrained in some way. The other areas of focus of Bowens framework [20] (adaptation, integration and expansion) were not chosen as this qualitative literature review is focussed on the feasibility of existing interventions and not assess the adaptation, integration and expansion of these interventions. As this study is based on qualitative findings, we also did not include the area of focus: limited-efficacy testing. In the analysis, we used the content in the qualitative result section as the meaningful units and they were read through several times to get familiar with them. A deductive approach goes from the areas of focus to observation to confirmation and often used when existing knowledge is used in a new context [21]. In our study, the existing knowledge was Bowens framework [20] of feasibility research. The new context was exergaming in older adults. The meaningful units were extracted and transferred to Nvivo and unconstrained categorization matrix were developed based Bowen´s [20] four areas of feasibility (Acceptability, Demand, Implementation & Practicality). All the meaningful units under each feasibility area were coded by LK and PR. [22] In the next step, subcategories were developed under each of the four areas of focus, following the principles of inductive content analysis [19]. The subcategories were discussed among all authors till agreement was reached. Effect sizes for each of the four areas of feasibility (categories) were calculated by the frequency of occurrence (number of articles divided by the total number of articles included in this literature review) for extracting more meaning from those data and verifying the presence of a category or subcategories, and to avoid the possibility of over or underweighting findings [23, 24].

## Results

### Studies Characteristics

In total 26 articles were included in this literature study including a qualitative design. Twelve studies were performed in Europe, seven articles included older adults in USA, three in Canada, two in Australia, one in Asia and one in Africa. Ten studies specifically included older adults with a disease; six studies included patients with stroke, two studies included patients with heart failure, one study included patients with multiple sclerosis, one study included patients with major neurocognitive disorder. Fourteen studies used a commercial exergame platform (e.g. Nintendo Wii or Microsoft Xbox360) and 12 studies developed or adapted an exergame (see Table 1). The literature study showed that most of the qualitative findings were based on content analysis. There were methodological shortcomings in the way the data analyses were presented in the articles included, and in many of the articles where quantitative and qualitative methods are used, the qualitative part functions more as an embellishment of the quantitative data. For further quality appraisal of the articles included in this literature review see supplement Table 1.

**Fig. 2:**
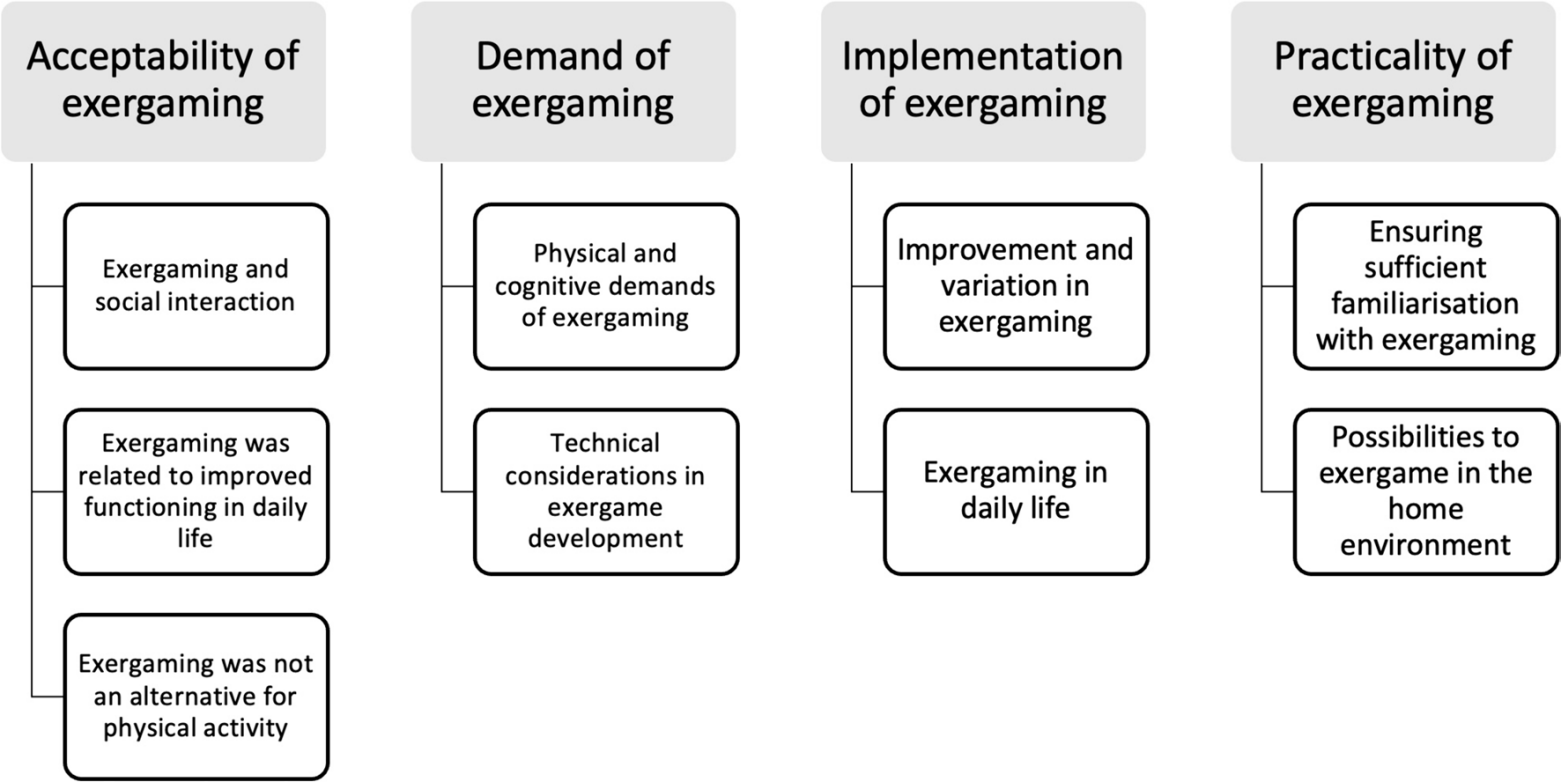
Categories and sub- categories for the feasibility of exergaming in older adults

The feasibility of exergaming in older adults was presented by four categories based on the areas in the framework for feasibility studies by Bowen [20]. The first category, acceptability of exergaming, had two subcategories: enjoyment and social interaction, and functioning in daily life. The second category, demand of exergaming had three subcategories: physical and cognitive demanding, technical challenges, and safety. The third category, implementation of exergaming had three subcategories: follow improvement and being challenged, exergaming in daily life and suitable for age group. The fourth category, practicality of exergaming had two subcategories: familiarization and home environment (see Fig. 2).

### Acceptability of Exergaming

Bowen defines acceptability as the reactions to the intervention, in this case the exergaming. Participants found exergaming enjoyable, especially with social interaction. Exergaming was seen a part of functioning in daily life. In total 23 studies included results on the category acceptability of exergaming (effect size 88%).

#### Exergaming and Social Interaction

Participants enjoyed exergaming [14, 25–43] and they liked it especially when playing with others (e.g. health care professionals, peers, spouses and grandchildren) [25, 26, 30, 31, 34–38, 44] or when exergaming included elements of competition [31, 34, 35, 37, 44]. Participants could for example play bowling together, where others cheered when throwing a strike, or expressed support when they had poor series [31, 35]. Social interaction during exergaming could also decrease confidence, as others might have higher scores or they could feel watched or judged [37]. Participants enjoyed the avatar in the exergame and could associate themselves with their avatar [35, 42].

#### Exergaming was Related to Improved Functioning in Daily Life

Some participants perceived that exergaming could improve their functioning in their daily life, [26, 36, 43, 45] increase their physical activity and balance [14, 25, 30, 32, 34, 35, 38, 43, 44, 46] and could increase cognitive function [14, 30, 38, 43, 44]. Exergaming was seen as a good addition to rehabilitation [31, 38].Participants could feel more confidence and empowered in performing their physical tasks and this made them aware that they could do more thinks that they initially assumed [36, 37, 46]. Exergaming was seen as a possible activity for promoting active ageing.

#### Exergaming was not an Alternative for Physical Activity

Participants could also experience exergaming as boring, especially over time, felt that exergaming did not bring enough exertion or even felt the exergames were patronizing [26, 28, 31, 33, 35, 37, 40–42]. Participants expressed that exergaming did not motivate them to be physically active [35, 45] and would rather participate in alternative exercise activities (e.g., aqua aerobics or outdoor activities) [14, 26, 35, 39, 43].

### Demand of Exergaming

Bowen defines demand for the exergaming as estimation of use and demand. Exergaming was seen as physical and cognitive demanding. There could be technical and safety challenges with exergaming. In total 25 studies included results on the category demands of exergaming (effect size 96%).

#### Physical and Cognitive Demands of Exergaming

Exergaming was experienced as safe [25, 26, 35, 36, 42, 47], although participants could experience difficulties in maintaining their balance, either to physical reasons, or because the floor was too slippery [25, 42].

Participants could experience exergaming as physically or cognitively demanding [25, 30, 37, 42, 45, 48], for example having muscle pain after or during exergaming [25, 30, 45]. Others experienced that exergaming made them more aware of their physical limitations [30, 39, 44, 46].

#### Technical Considerations in Exergame Development

Participants could experience technical challenges in exergaming [14, 25, 28, 29, 33, 41–45, 47]. Participants could get discouraged or frustrated, when they felt that the game was unnecessary complex. [14, 25, 26, 29, 30, 33, 39, 40, 48, 49] Especially when the exergame had visuals that were in fast pace, when they were unable to reach the targets in the exergame or when exergaming took complex motor tasks of simultaneously handling the controller while pressing the buttons and moving the arm [14, 25, 26, 30, 33, 42, 44, 48].

The participants requested that the exergaming should be more intuitive, such as a greeting message to inform the player of when the game was about to start [27, 43, 45]. A short-cut provided in the exergame (e.g. a home button to return to the beginning screen of the exergame) was seen important, as this enabled the participants to navigate easier and could help the acceptability of exergaming [25, 28, 43]. The possibility of music in the exergame and sounds indicating their game performance was seen as important, as this provided encouragement and entertainment value [27, 29, 42]. It was important for participants to be able to mute the music, as feedback provided during the songs could be experienced as distracting and not beneficial to performance [29, 38, 39]. Participants indicated that they liked summary feedback at the end of exergaming or (visual) feedback during exergaming, which should be included in exergaming [25, 26, 29, 30, 32, 34, 35, 37, 43].

### Implementation of Exergaming

Bowen defines implementation regarding the extent, likelihood and in which manner exergaming can be implemented as proposed or planned. To be able to follow improvement and being challenged in the exergame was seen as important to implementation. Participants expressed how to implement exergaming into their daily life.

In total 15 studies included results on the category implementation of exergaming (effect size 58%).

#### Improvement and Variation in Exergaming

Participants liked it when they could follow their exergame improvement, as they could be motivated to improve their scores [25, 26, 30, 32, 34, 35, 37, 39, 40, 42–44].With others mastery of the game and achieving improvement in the exergame did not provide further motivation to exergame [26, 35]. Participants expressed that variation in exergames would enhance their desire to play more [26, 27, 34, 35].

#### Exergaming in Daily Life

Important for implementation of exergaming in the participants life, was to find routines in exergaming [25, 35]. Participants that were exergaming for a couple of weeks, did find the exergaming more predictable, which made it harder to maintain exergaming [26, 31, 35, 43].

Participants found exergaming more realistic and therefore easier to implement into daily life when it involved movements that were movements related to everyday life activities or sports, for example walking or bowling [35, 44, 48].

### Practicality of Exergaming

According to Bowen practicality refers to how exergaming can be delivered when resources, time, and commitment are constrained in some way. A good familiarisation was seen as important when starting exergaming and it was seen as convenient when exergaming was possible in the home environment. In total 12 studies included results on the category practicality of exergaming. This category had the lowest effect size of 46%.

#### Ensuring Sufficient Familiarisation with Exergaming

The results show the importance to a good familiarisation to the exergame, preferable given by health care professionals, to be accepted by the participants as a mode to be physically active [25, 26, 35–37, 45]. It takes participant time to get comfortable to play and studies performed telephone follow-ups and even additional home visits to help the participants in starting exergaming [25, 26, 35, 37, 45] Participants like and needed to receive clear instructions how exergaming works, form either the game itself, on paper or videos or/and from a health care professional (e.g. occupational therapist or physical therapist) [30, 36, 37, 45].

#### Possibilities to Exergame in the Home Environment

Participants would like to have the possibility of exergaming in their own home environment [26, 28, 30, 34, 35, 37, 38]. Participants experienced that exergaming could reduce barriers to being active [14, 26, 35], for example the travel time needed to a treatment center when exergaming could be done at home [26, 28, 30, 34, 35, 37, 38, 43].

#### Practical Implications for Exergaming in Older Adults

For the practical implications for exergaming in older adults, we adapted the GameFlow model [54]. This model is designed for healthy adults and gaming, not exergaming in older adults. With the results of the literature review, we adapted this model and named it ExerGameFlow model for older adults (Table 2).

**Table 2:** ExerGameFlow model criteria for exergaming enjoyment in older adults

Element	Criteria
**Concentration** Exergames should require concentration and the players should be able to concentrate on the game	- Exergames must provide stimuli that are worth attending to - Players shouldn’t be burdened with tasks that don’t feel important - Exergames should have variation, while still being appropriate for the players physical and cognitive limitations - Music or sounds should be an available stimulus, but the possibility to mute and still be able to exergame should exist - Exergaming should be possible to do in the home environment
**Challenge** Exergames should be sufficiently challenging and match the players’ skill level	- Challenges in the exergames must match the older adults’ physical and cognitive levels - Exergames should provide different levels and variation of physical activity - The level of challenge and variation of the exergame should increase as the older adults progresses - Exergames should provide new challenges at an appropriate pace
**Player Skills** Exergames must support player skill development and mastery	- Exergame interfaces and mechanics should be easy to learn and use - A good familiarisation to the exergame is important, either within the exergame, on paper or through videos or given personally - The surrounding of the physical activity performed with the exergame should be safe (e.g. no slippery floors) - Follow-up after familiarisation (e.g. telephone follow-ups) should be given to motivate the players to be physically active - Exergames should increase the players’ skills at an appropriate pace as they progress through the exergame - Players should be rewarded during exergaming for their effort and skill development
**Control** Players should feel a sense of control over their actions in the exergame	- Players should feel a sense of control over their avatar or units and their movements and interactions in and with the exergame - Players should feel a sense of control over the exergame interface and input devices - Players should feel a sense of control over the game shell (starting, stopping, saving, etc.). A short-cut should be provided in the exergame, as this enables the players to navigate easy - Players should not be able to make errors that are detrimental to the exergame and should be supported in recovering from errors
**Clear Goals** Exergames should provide the player with clear goals	- Exergames should include clear goals in the game and on the players physical activities which they must perform
**Feedback** Players must receive appropriate feedback at appropriate times	- Players should receive feedback on the progress in the exergame and the progress of their physical activity - A summary of the exergame results and the results of the physical activity performed should be given at the end of each session - Players should always be able to access their physical activity status or score
**Immersion** Players should experience effortless involvement in the game	- Exergames physical activity should be closely related to everyday life activities or sports
**Social Interaction** Exergames should have the possibility to play together with others	- Exergaming should have the possibility to play with others or to compete within the exergame or with others - Exergames should have the ability to support social communities inside and outside the game

## Discussion

This literature review provides implication on the acceptability of exergaming in older adults, the demands of exergaming, the implementation, and practicalities and gave practical implication for the development of exergames and exergame interventions for older adults.

Enjoyment in exergaming, especially when social playing is possible, and functioning in daily life were seen by older adults important for the acceptability of exergaming. Other studies show that if participants feel enjoyment during the physical activity, they increase the amount of physical activity and even are more adherent to physical activity recommendations [50, 51]. Also the adherence to technology driven exercise is higher due to enjoyment [52] and in gaming, player enjoyment is seen as the goal for a successful game development, as players who do not experience enjoyment will not play the game [53, 54]. Social interaction is a crucial aspect of computer games for older individuals [55–57]. Therefore, exergames should enable multiplayer options for playing together or competing. Furthermore, exergaming should foster social communities both within and beyond the game environment.

Exergaming could be experienced as physical and cognitive demanding, and some technical challenges could occur. As also other studies suggests, exergames should match older adults' skill levels and preferences for challenging activities [58, 59]. Exergames for older adults should support skill development with easy-to-use interfaces and clear instructions [60]. Studies show that older adults typically prefer puzzle and strategy games that are easy to learn but provide a challenge [59]and these could be considered in designing exergames for older adults.

While audio elements can enrich the experience, our study show that providing a mute option is beneficial. Incorporating shortcuts can aid navigation. Older adults should feel empowered and in control while exergaming, overseeing their avatars' actions and interactions. They should have autonomy over basic functions like starting or saving, with the game design preventing errors and providing support for recovery if needed.

Safety measures are crucial, requiring hazard-free environments. The physical hardware must ensure safety by preventing tripping and falling, being easily accessible for people with physical disabilities, and simple to sanitize [27, 61–63].

For the implementation of exergaming, it is important that they can follow improvement and that they are being challenged. This literature review shows how older adults implement exergaming in their daily life. Exergames should have clear, meaningful goals aligned with players' motivations and interests, guiding both in-game actions and associated physical activities for older adults [64–66]. Older adults should receive continuous feedback on both their in-game progress and physical activity levels, with comprehensive summaries provided after each session. This direct feedback enhances the exergame experience, making it more rewarding. Older adults should experience effortless involvement in the exergame, which should closely relate to everyday activities or sports. Immersion serves as a key motivator for retention [67].

For the practicalities of exergaming a good familiarisation was seen as important and the possibility to exergame at home. Post-familiarization follow-ups are recommended for sustained engagement. As also shown in other studies, exergaming should ideally be adaptable for home use, with game design enhancing concentration [68, 69].

### Methodological Considerations

We used available qualitative methodological checklists for evaluating the quality of the studies included in the review and analysed the results of the included articles using an established method. The articles included in this literature review limiting to English, which may have excluded some relevant literature. In addition, we choose to use four of the seven areas of focus of feasibility (acceptability, demand, implementation and practicality) [20] for deductive analysis, as this qualitative literature review is focussed on the feasibility of existing interventions and not assess the adaptation, integration and expansion of exergaming. Although, it is likely that the four areas chosen, described the feasibility of exergaming in older adults, identifying adaptation, integration and expansion of exergaming could have provided additional understanding of exergaming in older adults.

### Future Research

To develop and have a deeper understanding of exergaming for older people, well-designed qualitative studies are needed that can stand on their own and provide reliable qualitative data. The results of this literature review can provide guidance for the future development and testing of exergames for older adults and exergame interventions.

## Conclusion

In conclusion, exergaming presents promising to increase physical activity among older adults. Based on the experience of older adults, not only did they found exergaming enjoyable, but it also offers cognitive and physical challenges that can enhance overall well-being. However, navigating technical and safety challenges requires careful consideration and support. Thorough familiarization with written and video instructions, ongoing assistance, and ensuring home accessibility are important in successfully introducing exergaming to older adults. Moreover, the importance to be able to follow improvement and tailoring challenges to suit individual needs should be considered. By addressing these factors, exergaming could be a valuable tool in promoting active and healthy aging among older adults.

### Supplementary Information

Below is the link to the electronic supplementary material.

Supplementary file1 (DOCX 20 KB)
