# Supplementary material for: Exergaming to Increase Physical Activity in Older Adults: Feasibility and Practical Implications
Source: Curr Heart Fail Rep. 2024 Jul 18;21(4):439–59. doi: 10.1007/s11897-024-00675-9 (PMC11333506; doi:10.1007/s11897-024-00675-9)
Supplement: Supplementary file 1 — Supplementary file1 (DOCX 20 KB) [file 11897_2024_675_MOESM1_ESM.docx]

Supplement table 1 Quality appraisal of the articles included in the literature review on feasibility of exergaming in older adults.

|  | Authors, Year of publishing | Clear statement of the aims of the research | Methodology appropriate | Research design appropriate to address the aims of the research | Recruitment strategy appropriate to the aims of the research? | Data collected in a way that addressed the research issue? | The relationship between researcher and participants been adequately considered | Ethical issues been taken into consideration | The data analysis was sufficiently rigorous | Clear statement of findings | Valuable |
| --- | --- | --- | --- | --- | --- | --- | --- | --- | --- | --- | --- |
| 1 | Agmon et al, 2011, | Yes | No | Yes | Yes | Yes | No | Yes | No | Yes | Yes |
| 2 | Barg-Walkow et al, 2017 | Yes | No | Yes | Yes | Yes | No | No | No | Yes | Yes |
| 3 | Brown et al., 2015 | Yes | Yes | Yes | Yes | Yes | No | Yes | No | Yes | Yes |
| 4 | Cacciata et al., 2021 | Yes | Yes | Yes | Yes | Yes | No | Yes | Yes | Yes | Yes |
| 5 | Celinder et al., 2012 | Yes | Yes | Yes | Yes | Yes | No | Yes | No | Yes | Yes |
| 6 | Cao et al., 2016 | Yes | Yes | Yes | Yes | Yes | No | Yes | Yes | Yes | Yes |
| 7 | Chu et al., 2021 | Yes | Yes | Yes | Yes | Yes | Yes | Yes | Yes | Yes | Yes |
| 8 | Demers et al., 2018 | Yes | Yes | Yes | Yes | Yes | Yes | Yes | Yes | Yes | Yes |
| 9 | De Vries et al., 2018 | Yes | Yes | Yes | Yes | Yes | No | Yes | Yes | Yes | Yes |
| 10 | Ellmers et al., 2018 | Yes | Yes | Yes | Yes | Yes | No | Yes | Yes | Yes | Yes |
| 11 | Finley & Combs, 2013 | Yes | No | No | Yes | Yes | No | No | No | Yes | Yes |
| 12 | Forsberg et al., 2015 | Yes | Yes | Yes | Yes | Yes | Yes | Yes | Yes | Yes | Yes |
| 13 | Freed et al., 2021 | Yes | Yes | Yes | Yes | Yes | No | Yes | No | Yes | Yes |
| 14 | Howes et al., 2021 | Yes | Yes | Yes | Yes | Yes | No | Yes | Yes | Yes | Yes |

|  | Authors, Year of publishing | Clear statement of the aims of the research | Methodology appropriate | Research design appropriate to address the aims of the research | Recruitment strategy appropriate to the aims of the research? | Data collected in a way that addressed the research issue? | The relationship between researcher and participants been adequately considered | Ethical issues been taken into consideration | The data analysis was sufficiently rigorous | Clear statement of findings | Valuable |
| --- | --- | --- | --- | --- | --- | --- | --- | --- | --- | --- | --- |
| 15 | Glännfjord et al., 2017 | Yes | Yes | Yes | Yes | Yes | Yes | Yes | Yes | Yes | Yes |
| 16 | Klompstra et al., 2017 | Yes | Yes | Yes | Yes | Yes | No | Yes | Yes | Yes | Yes |
| 17 | Koh et al., 2020 | Yes | Yes | Yes | Yes | Yes | No | Yes | No | Yes | Yes |
| 18 | Meekes & Stanmore, 2017 | Yes | Yes | Yes | Yes | Yes | Yes | Yes | Yes | Yes | Yes |
| 19 | Millington, 2015 | Yes | Yes | Yes | Yes | Yes | No | No | No | Yes | Yes |
| 20 | Money et al., 2019 | Yes | Yes | No | Yes | Yes | No | Yes | No | Yes | Yes |
| 21 | Rand et al., 2018 | Yes | Yes | Yes | Yes | Yes | No | Yes | Yes | Yes | Yes |
| 22 | Rogers et al., 2021 | Yes | Yes | Yes | Yes | Yes | No | Yes | No | Yes | Yes |
| 23 | Swinnen et al., 2021 | Yes | Yes | No | Yes | Yes | No | Yes | Yes | Yes | Yes |
| 24 | Tabak et al., 2020 | Yes | Yes | No | Yes | Yes | No | Yes | Yes | Yes | Yes |
| 25 | Valenzuela et al., 2018 | Yes | Yes | Yes | Yes | Yes | Yes | Yes | Yes | Yes | Yes |
| 26 | Vaziri et al., 2016 | Yes | Yes | Yes | No | No | No | No | No | Yes | Yes |
